# The *eta7/csn3-3* Auxin Response Mutant of Arabidopsis Defines a Novel Function for the CSN3 Subunit of the COP9 Signalosome

**DOI:** 10.1371/journal.pone.0066578

**Published:** 2013-06-07

**Authors:** He Huang, Marcel Quint, William M. Gray

**Affiliations:** Department of Plant Biology, University of Minnesota, St. Paul, Minnesota, United States of America; Iwate University, Japan

## Abstract

The COP9 signalosome (CSN) is an eight subunit protein complex conserved in all higher eukaryotes. In *Arabidopsis thaliana*, the CSN regulates auxin response by removing the ubiquitin-like protein NEDD8/RUB1 from the CUL1 subunit of the SCF^TIR1/AFB^ ubiquitin-ligase (deneddylation). Previously described null mutations in any CSN subunit result in the pleiotropic *cop/det/fus* phenotype and cause seedling lethality, hampering the study of CSN functions in plant development. In a genetic screen to identify enhancers of the auxin response defects conferred by the *tir1-1* mutation, we identified a viable *csn* mutant of subunit 3 (CSN3), designated *eta7/csn3-3*. In addition to enhancing *tir1-1* mutant phenotypes, the *csn3-3* mutation alone confers several phenotypes indicative of impaired auxin signaling including auxin resistant root growth and diminished auxin responsive gene expression. Unexpectedly however, *csn3-3* plants are not defective in either the CSN-mediated deneddylation of CUL1 or in SCF^TIR1^-mediated degradation of Aux/IAA proteins. These findings suggest that *csn3-3* is an atypical *csn* mutant that defines a novel CSN or CSN3-specific function. Consistent with this possibility, we observe dramatic differences in double mutant interactions between *csn3-3* and other auxin signaling mutants compared to another weak *csn* mutant, *csn1-10*. Lastly, unlike other *csn* mutants, assembly of the CSN holocomplex is unaffected in *csn3-3* plants. However, we detected a small CSN3-containing protein complex that is altered in *csn3-3* plants. We hypothesize that in addition to its role in the CSN as a cullin deneddylase, CSN3 functions in a distinct protein complex that is required for proper auxin signaling.

## Introduction

The phytohormone auxin (indole-3-acetic acid or IAA) regulates numerous plant developmental processes, by modulating the expression of auxin responsive genes to control cell division, expansion and differentiation [1]. Two large families of transcriptional regulators play essential roles in auxin mediated gene expression: short-lived Aux/IAA proteins and Auxin Response Factor (ARF) transcription factors. In the absence of an auxin signal, Aux/IAAs heterodimerize with ARFs to repress the transcriptional activation of auxin responsive genes [2], [3]. To activate auxin responsive gene expression, Aux/IAA repression must be removed, which is accomplished by the SCF^TIR1/AFB^ E3 ubiquitin ligase and the 26S proteasome mediated proteolysis of Aux/IAAs in an auxin-dependent manner [4]–[6].

Ubiquitin is a small conserved protein, which can be covalently conjugated to specific substrate proteins through the activity of a ubiquitin-activating enzyme (E1), a ubiquitin-conjugating enzyme (E2), and a ubiquitin-ligase (E3) [7]. Proteins with a polyubiquitin chain can then be recognized and degraded by the 26S proteasome. A major class of E3 ubiquitin ligases are the cullin-RING ligases (CRLs), among which the SCF (for Skp1-Cullin-F-box) sub-type has been well-studied [8]. SCF ligases consist of four subunits: the cullin protein, CUL1, binds the RING box protein RBX1 with its C-terminus, while the SKP1 adaptor protein (ASK1 in Arabidopsis) recruits any of many F-box proteins (FBPs) to the N-terminus of CUL1 [9], [10]. The TIR1, AFB1, AFB2, and AFB3 F-box proteins of the SCF^TIR1/AFB^ complex are auxin receptors, targeting Aux/IAAs for ubiquitination and degradation upon binding auxin [11]–[13]. Mutations in SCF^TIR1/AFB^ subunits, such as *axr6/cul1*, *ask1* and *tir1*, cause the stabilization of Aux/IAAs and a variety of auxin response defects including auxin resistant root growth and reduced lateral root development [4], [14], [15]. The proper function of SCF ligases, as well as other CRLs, also requires a process called neddylation, in which the ubiquitin-like protein RUB/NEDD8 is covalently conjugated to the cullin subunit [16]–[18]. The AXR1, ECR1, and RCE1 enzymes mediate cullin neddylation, and mutations in these factors result in a reduction in the level of neddylated cullin and pleiotropic growth defects, including diminished auxin signaling [16], [19]. The NEDD8 modification of CUL1 is a highly dynamic process [20], [21]. Cleavage of NEDD8 from cullins (deneddylation) is promoted by a protein complex called the COP9 signalosome (CSN), as the CSN complex purified from porcine spleen deneddylates Cul1 *in vitro* [21], [22]. Consistently, studies in various organisms have shown that neddylated cullin proteins accumulate in *csn* mutants [22]–[25].

The COP9 signalosome (CSN) is an evolutionarily conserved protein complex of eight subunits [26]. It was originally identified in Arabidopsis through genetic screens for mutants exhibiting a *co*nstitutive *p*hotomorphogenic/*d*e*-et*iolated *(cop/det*) phenotype, and was subsequently biochemically purified from both plant and animal protein extracts [26], [27]. The CSN is structurally related to the 19S lid of the 26S proteasome and eukaryotic translation initiation factor 3 (eIF3), and is composed of six PCI (for Proteasome, COP9, eIF3) domain-containing subunits (CSN1-4, 7, and 8) and two MPN (for Mpr1p, Pad1p N-terminal) domain-containing subunits (CSN5 and CSN6) [27]. It has been established that the CSN regulates SCF activity by deneddylating the CUL1 subunit of the SCF [22], [28]. Besides CUL1, the CSN also deneddylates other cullin proteins and broadly regulates many CRLs, including those containing CUL2, CUL3 and CUL4 [21], [23], [29], [30]. In plants, the CSN has been implicated in a variety of processes, including auxin, jasmonate (JA), and gibberellin (GA) signaling, flower development, and light signaling via its interaction with many CRLs [29], [31], [32]. CSN-mediated cullin deneddylation is catalyzed by the JAMM metallozyme motif within the CSN5 subunit [22], [33]. However, CSN5 can only provide this isopeptidase activity after incorporation into the CSN holocomplex, as deneddylation activity is defective in all previously characterized CSN subunit mutants [23], [34], [35].

Neddylation plays a positive role in regulating SCF activity, by promoting a conformational change in CUL1 that shortens the distance between FBP-bound substrates and the E2 ubiquitin-conjugating enzyme [36]–[38]. Although *in vitro* biochemical studies indicated that the CSN negatively regulates E3 ubiquitin ligase activity [21], [33], genetic evidence suggests otherwise [28], [39], [40]. Studies in yeast and *Drosophila* suggested that although the CSN returns CRL activities to basal levels, it can also facilitate CRL activities by either maintaining the stability of labile substrate adapters or by recycling unstable, neddylated cullins into the more stable deneddylated isoform [41], [42]. In Arabidopsis, the CSN is also required for the proper functions of SCF^TIR1/AFB^, as well as SCF^SLY1^, in the degradation of Aux/IAA proteins or the DELLA proteins, respectively [28], [31], [43]. Together, these findings suggest that cullin neddylation/deneddylation is a highly dynamic process essential for maintaining proper CRL function [44], [45]. In addition to regulating SCF activity by deneddylating cullins, recent biochemical findings indicate that the CSN also inhibits SCF complexes by a noncatalytic mechanism [46], [47]. Following deneddylation, the CSN remains stably associated with the SCF, sterically hindering both the re-neddylation of CUL1 and SCF interactions with the E2 enzyme.

Arabidopsis *null* mutants of any CSN subunit are phenotypically indistinguishable and exhibit the pleiotropic *cop/det/fus* phenotype, which is characterized by short hypocotyl and open cotyledons of dark-grown seedlings, accumulation of anthocyanin and seedling lethality [23], [27], [48], [49]. Severe deneddylation defects have been found in previously described null mutants of each of the CSN subunits [23], [48], but the associated seedling lethality hampers the further analyses of CSN functions. Recently however, a few weak, viable Arabidopsis *csn* mutants have been described. These include mutants lacking one of the two copies of *CSN5* and *CSN6* encoded in the Arabidopsis genome [23], [50], as well as hypomorphic missense alleles of *CSN1* and *CSN2* [40], [43]. Importantly however, these viable *csn* mutants still exhibit diminished deneddylation activity, resulting in the accumulation of neddylated CUL1 and reduced SCF activity [23], [28], [34], [35], [40], [43].

In a previously described genetic screen for enhancers of *tir1-1* [15], [51]–[54], we identified two weak *csn* subunit mutants, designated as *eta6/csn1-10* [43] and *eta7/csn3-3*. Our phenotypic characterization of these *csn* mutants, together with expression studies with auxin regulated reporters, demonstrate that *csn1-10* and *csn3-3* confer very similar reductions in auxin response. However, unlike *csn1-10*, which is a typical *csn* mutant with defects in CSN-mediated deneddylation and Aux/IAA protein degradation [43], neither of these defects were observed in the *csn3-3* mutant. Furthermore, genetic interactions between these *csn* mutants and additional auxin signaling mutants also distinguish *csn3-3* from other *csn* mutants. Our studies suggest that *csn3-3* is a unique *csn* mutant that defines a novel functional activity for the CSN3 subunit of the COP9 signalosome in the regulation of auxin signaling.

## Results

### *eta7* is a weak allele of COP9 signalosome subunit 3 (CSN3)

**Figure 1:**
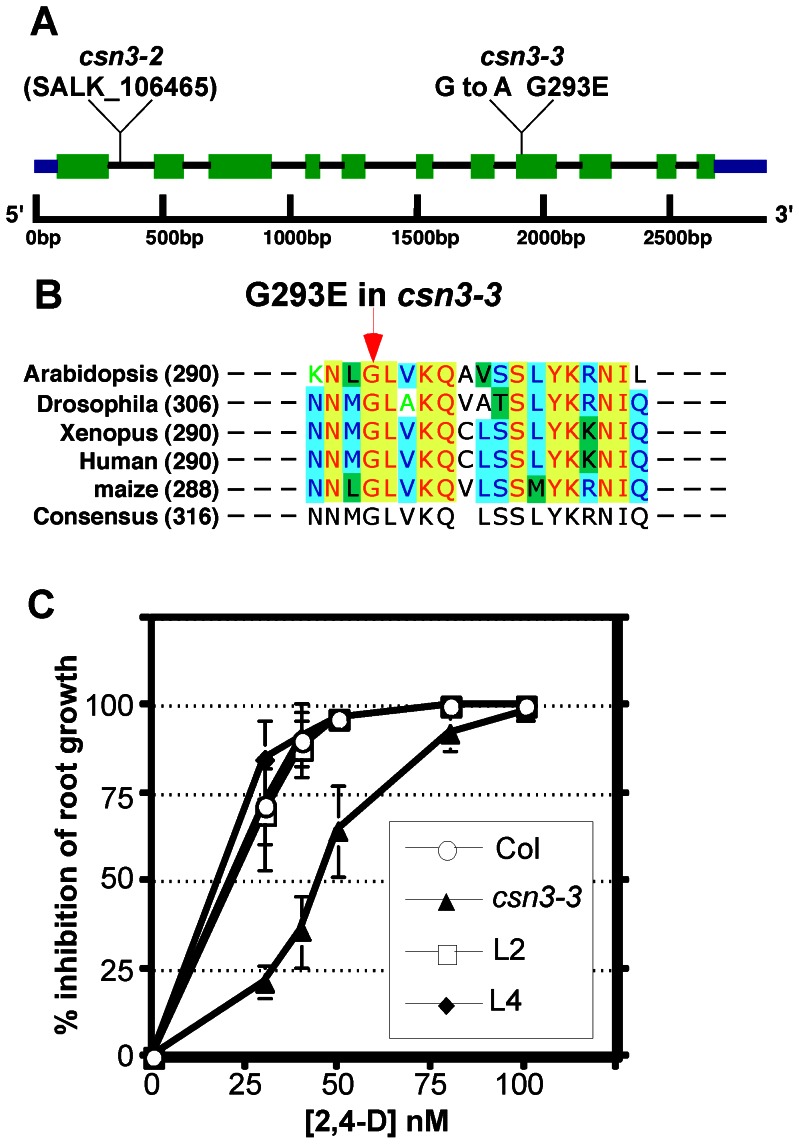
*csn3-3* is a weak missense allele of *CSN3*. (**A**) Schematic representation of the Arabidopsis *CSN3* locus. Positions of the *csn3-3* missense mutation and previously described *csn3-2* T-DNA insertion are indicated. (**B**) Sequence alignment around the *csn3-3* mutation among the CSN3 homologs from *Arabidopsis thaliana*, *Drosophila melanogaster*, *Xenopus laevis*, *Homo sapiens* (Human) and *Zea mays* (maize). The arrow indicates the position of the *csn3-3* G293E missense mutation. (**C**) Complementation of the *csn3-3* auxin resistance phenotype by introduction of a genomic CSN3 transgene. L2 and L4 are two independent *csn3-3*[*gCSN3*] transformants.

We have previously reported the identification of several auxin response mutants isolated from a genetic screen for *e*nhancers of the *tir1-1 a*uxin response defect (designated as *eta* mutants), including *eta1/axr6-3* [15], *eta2-1/cand1* [52], *eta3/sgt1b* [53], *eta4/pdr9-1* [54], *eta5/iar4* [51] and *eta6/csn1-10* [43]. The *eta7* mutant was also identified in this screen. Map based cloning positioned the *eta7* mutation within a 330 kb interval on chromosome five. This interval contained 102 predicted genes, including *CSN3/FUS11*, which encodes subunit 3 of the COP9 signalosome (CSN) [55]. Given that *csn* subunit mutations are known to confer diminished auxin response phenotypes, we sequenced the *CSN3* locus from *eta7* plants and identified a single mutation (**Figure 1A**). This mutation results in a G293E missense mutation within the highly conserved PCI domain of CSN3. This domain is important for subunit interaction and CSN complex assembly [56]. Primary sequence alignment among the PCI domains of several CSN3 orthologs revealed that Gly293 is extremely highly conserved throughout eukaryotes (**Figure 1B**). To confirm that this mutation was responsible for the *eta7* auxin response defect, we conducted complementation tests by transforming a genomic *CSN3* construct into *eta7* mutant plants. The *CSN3* transgene fully restored auxin sensitivity to *eta7* seedlings when tested in root growth assays (**Figure 1C** **)**. We therefore renamed *eta7* as *csn3-3*.

### Characterization of *csn3-3* phenotypes

**Figure 2:**
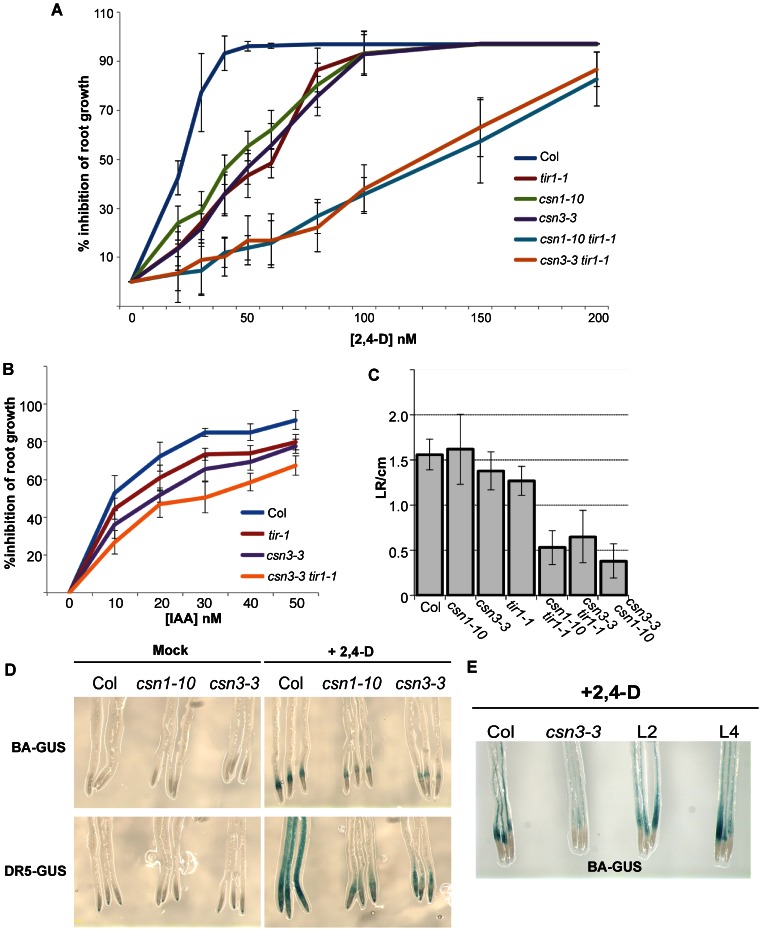
*csn3-3* confers auxin response defects. (**A**) Inhibition of root elongation by increasing concentrations of the synthetic auxin 2,4-D. 5-day-old (d.o.) seedlings (*n*≥15) grown on ATS medium were transferred to medium containing 2,4-D and grown for another four days. Data are presented as percent inhibition of root growth compared to growth on unsupplemented ATS. Error bars = SD. (**B**) IAA dose-response curve of inhibition of root growth. (**C**) Lateral root (LR) initiation was measured in 11-d.o. seedlings grown on unsupplemented ATS medium. Data were presented as number of the lateral root initiations per cm root length. Error bars = SD (*n*≥15). (**D**) Transgenic Col, *csn1-10* and *csn3-3* carrying either the *BA3*:*GUS* or *DR5*:*GUS* reporters. 6-d.o. seedlings were treated with 0.5 µM 2,4-D for 12 h (*BA3*:*GUS*) or 4 h (*DR5*:*GUS*) before histochemical staining for β-glucuronidase activity. (**E**) Complementation of the reduced *BA3:GUS* expression phenotype of *csn3-3* seedlings by a genomic *CSN3* transgene. L2 and L4 are two independent *csn3-3*[*gCSN3*] transformants.

The CSN plays a well-established role in auxin signaling, acting as a deneddylase to regulate SCF^TIR1/AFB^ activity. Mutants in any CSN subunit exhibit auxin related phenotypes, such as auxin resistant root growth and reduced lateral root formation [28], [34], [43]. In a dose-response assay measuring the auxin inhibition of root elongation, we found *csn3-3* was mildly resistant to exogenous auxins. After transfer to media supplemented with 0.05 µM 2,4-D, root growth of wild-type (Col) seedlings was nearly completely inhibited (**Figure 2A**). However, *csn3-3* seedlings were resistant to this inhibition, displaying only 47% inhibition of root growth. A similar degree of auxin resistant root growth was observed with the *csn1-10* and *tir1-1* mutants. Additionally, both *csn1-10* and *csn3-3* enhanced the weak auxin resistance phenotype of *tir1-1*, with *csn1-10 tir1-1* and *csn3-3 tir1-1* double mutants exhibiting comparable dose-response profiles in root growth inhibition assays (**Figure 2A**). Similar assays using IAA-supplemented media also found that *csn3-3* exhibited auxin resistance and enhanced the *tir1-1* phenotype, demonstrating that the auxin response defect of *csn3-3* is not 2,4-D specific (**Figure 2B**).

Previous reports have shown that plants expressing a *CSN5* antisense construct develop fewer lateral roots and reduced root hair elongation compared to wild-type controls [28]. Consistent with this finding, the *csn3-3* and the *csn1-10* mutations enhanced the weak lateral root defect of *tir1-1* seedlings, with both double mutants developing fewer than 50% of the number of lateral roots observed in *tir1-1* seedlings (**Figure 2C**). Furthermore, while lateral root numbers in *csn3-3* and *csn1-10* single mutants were comparable to wild-type controls, *csn3-3 csn1-10* double mutants developed significantly fewer lateral roots, suggesting that auxin response is more severely impaired in the double mutant background.

To further compare the auxin response defects of *csn3-3* and *csn1-10* mutants, we introduced the *BA3:GUS* and *DR5:GUS* auxin responsive reporters [3], [57] to examine auxin mediated gene expression. Treatment of Col [*BA3:GUS*] control seedlings with 2,4-D or IAA triggered a strong GUS signal in the root elongation zone. In contrast, only a slight induction of *BA3:GUS* activity was observed in *csn3-3* and *csn1-10* mutants (**Figures 2D and S** **1**). Like the auxin-resistant root growth phenotype of *csn3-3*, the diminished *BA3:GUS* expression was complemented by a genomic *CSN3* transgene (**Figure 2E**). Similar findings were obtained with *csn3-3* and *csn1-10* seedlings carrying the *DR5:GUS* reporter (**Figure 2D**). Together, our analysis of root growth inhibition, lateral root development, and auxin mediated gene expression suggest that the *csn3-3* and *csn1-10* mutations confer a very similar reduction in auxin sensitivity.

**Figure 3:**
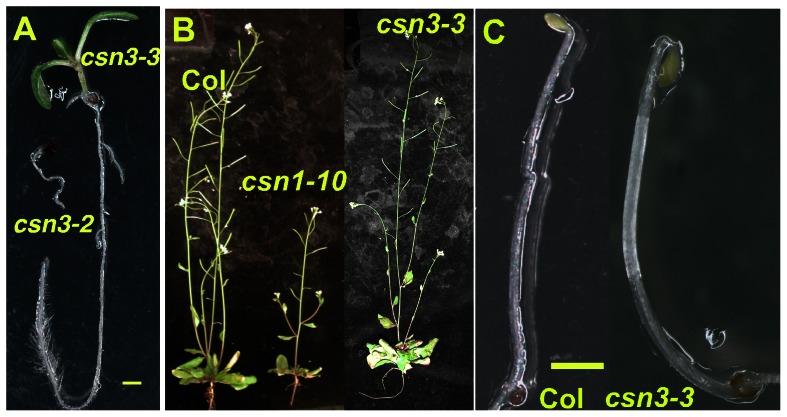
*csn3-3* does not exhibit characteristic ***csn*** mutant phenoytpes. (**A**) Seedling phenotypes of *csn3-2* and *csn3-3* mutants. (**B**) Phenotypes of Col, *csn1-10* and *csn3-3* adult (30-d.o.) plants. (**C**) 5-d.o. Col and *csn3-3* etiolated seedlings. Size bars = 1 mm.

In Arabidopsis, loss of any of the eight CSN subunits results in an identical suite of phenotypes, including constitutive photomorphogenesis, anthocyanin accumulation, and seedling lethality [23], [35], [58]. We observed similar phenotypes upon examining the *csn3-2* null mutant (**Figure 3A**). Previously described viable *csn* mutants include single mutants of the two MPN domain subunits, which are each encoded by two highly homologous genes (*CSN5A/CSN5B* [59] and *CSN6A/CSN6B* [50]), and the weak *csn1-10* [43] and *csn2-5* [40] missense mutants. Unlike previously described *csn3* alleles, [23], [35], [55], *csn3-3* is viable throughout development and does not exhibit a constitutive photomorphogenic (*cop*) phenotype (**Figure 3A-C**). Likewise, the *csn1-10* and *csn2-5* mutants also do not exhibit an obvious *cop* phenotype, although both mutants are mildly dwarfed compared to wild-type and *csn3-3* adult plants [40], [43].

### CUL1 deneddylation and Aux/IAA stability are not affected in *csn3-3* seedlings

**Figure 4:**
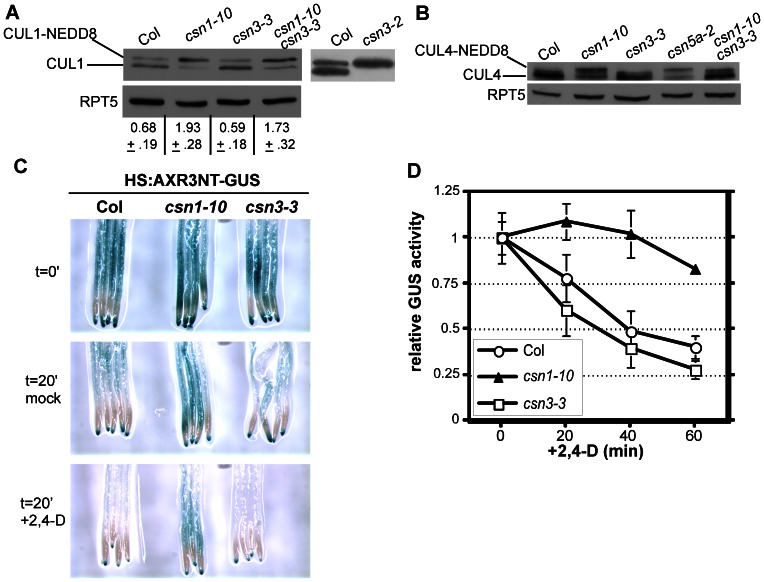
*csn3-3* affects auxin response independent of SCF^TIR1/AFB^. (**A**) CUL1 western blot analysis of protein extracts prepared from Col and *csn* mutant seedlings. The upper band indicates the modified (neddylated) CUL1. RPT5 is shown as a loading control. Numbers below the blot indicate the ratio of CUL1-NEDD8:CUL1 ± SD from three experiments. (**B**) Western blot analysis of CUL4 neddylation status in Col and *csn* mutant seedling extracts. (**C**) Col, *csn1-10* and *csn3-3* carrying the *HS:AXR3NT-GUS* transgene were heat shocked for 2 h and stained immediately or following incubation with 1 µM 2,4-D for 20 min. (**D**) Quantitative measurement of the β-glucuronidase activity of the *HS:AXR3NT-GUS* reporter in Col, *csn1-10*, and *csn3-3* seedlings. Seedlings were heat-shocked for 2 h, and then returned to room temperature and treated with 1 µM 2,4-D for 20, 40, or 60 min. β-glucuronidase activity is presented as the fraction remaining compared to the 0 min time point. Values shown depict the mean ± SD of 6 technical replicates. Similar results were obtained in two additional biological replicates.

The CSN cleaves the RUB/NEDD8 peptide from the cullin subunit of CRL ubiquitin-ligases. All previously described *csn* mutants, including the weak *csn1-10* and *csn2-5* alleles, result in an increase in the CUL1-NEDD8 to unmodified CUL1 ratio [40], [43]. We therefore examined if the *csn3-3* mutation affected CUL1 modification. We included *csn1-10* for comparison with *csn3-3*, since both mutants are viable *csn* mutants and display similar auxin response defects (**Figure 2** **)**. While a clear increase in CUL1-NEDD8:CUL1 was seen in extracts prepared from *csn1-10* seedlings, no change from wild-type was detected in α-CUL1 western blots of *csn3-3* extracts (**Figure 4A**). In contrast, CUL1 was exclusively in its neddylated form (CUL1-NEDD8) in *csn3-2* extracts (**Figure 4A**), as reported in previous studies [35].

Since the *csn3-3* mutation unexpectedly did not affect the CUL1-NEDD8:CUL1 ratio, we examined double mutants with *csn1-10* to test the possibility that *csn3-3* might enhance the weak deneddylation phenotype of *csn1-10*. Once again however, the *csn3-3* mutation did not increase the CUL1-NEDD8:CUL1 ratio (**Figure 4A**). Since the CSN also regulates other cullin-based E3 ubiquitin-ligases by deneddylating their cullin subunit, we applied a similar immunoblotting assay to examine CUL4 modification in *csn3-3* seedlings. Similar to our findings with CUL1, we observed no accumulation of the CUL4-NEDD8 isoform in *csn3-3* extracts, whereas neddylated CUL4 was readily detectable in *csn1-10* and *csn5a-2* extracts (**Figure 4B** **)**. Together, our analyses of CUL1 and CUL4 suggest that the *csn3-3* mutation does not affect the deneddylation activity of the CSN.

The reduced deneddylation activity of several previously characterized *csn* mutants has been found to result in diminished SCF^TIR1/AFB^ activity, resulting in the stabilization of Aux/IAA proteins [28], [40]. Since our finding that *csn3-3* plants exhibited no change in cullin deneddylation was quite surprising, we examined SCF^TIR1/AFB^ activity by monitoring Aux/IAA stability using the previously described HS:AXR3NT-GUS reporter protein [4]. *csn1-10* seedlings were again included for comparison. 6-d.o. Col, *csn1-10* and *csn3-3* seedlings, all of which carried the *HS:AXR3NT-GUS* construct, were heat-shocked at 37°C for 2 hours to induce expression, followed by return to ambient temperature and treatment with auxin. AXR3NT-GUS activity was examined both qualitatively and quantitatively at 20 min intervals during the treatment to monitor the remaining levels of AXR3NT-GUS fusion protein (**Figure 4C, D** **)**. Following the 2h heat-shock induction, similar AXR3NT-GUS levels were observed in wild-type, *csn3-3* and *csn1-10* seedlings (**Figure 4C**). During the auxin treatment, AXR3NT-GUS activity in *csn3-3* and wild-type seedlings diminished with comparable kinetics (**Figure 4D** **)**, suggesting that SCF^TIR1/AFB^ activity is unaffected in *csn3-3* plants. In contrast, *csn1-10* seedlings exhibited substantially slower AXR3NT-GUS degradation (**Figures 4C, D**).

To further confirm that *csn3-3* had no effect on SCF^TIR1/AFB^-mediated proteolysis, we examined another Aux/IAA reporter, IAA28-myc [60]. The IAA28-myc construct was crossed into *csn3-3* and *csn1-10* backgrounds. Abundance of the IAA28-myc protein was then examined by treating seedlings with IAA and immunoblotting root protein extracts. As previously reported [60], IAA28-myc was rapidly degraded in wild-type seedlings and was nearly undetectable after a 10 minute auxin treatment (**Figure S2**). While IAA28-myc was clearly more stable in *csn1-10* seedlings, it was rapidly degraded in the *csn3-3* background (**Figure S2**). Combined with our HS:AXR3NT-GUS degradation data, this strongly suggests that SCF^TIR1/AFB^ activity is unaltered by the *csn3-3* mutation.

### Genetic interactions distinguish *csn3-3* and *csn1-10*

The CSN regulates auxin signaling by deneddylating CUL1 to modulate SCF^TIR1/AFB^ activity [22], [28], [44]. Our findings with the *csn1-10* mutant are consistent with this model. The *csn3-3* mutation on the other hand, affects neither CUL1 deneddylation nor SCF^TIR1/AFB^-mediated Aux/IAA degradation, yet confers auxin response defects virtually identical in severity to *csn1-10*. These findings strongly suggest that the CSN, or at least the CSN3 subunit, plays a second role in the regulation of auxin signaling in addition to deneddylating CUL1. If so, we reasoned that *csn3-3* and *csn1-10* may exhibit distinct genetic interactions when combined with other mutations affecting auxin signaling.

**Figure 5:**
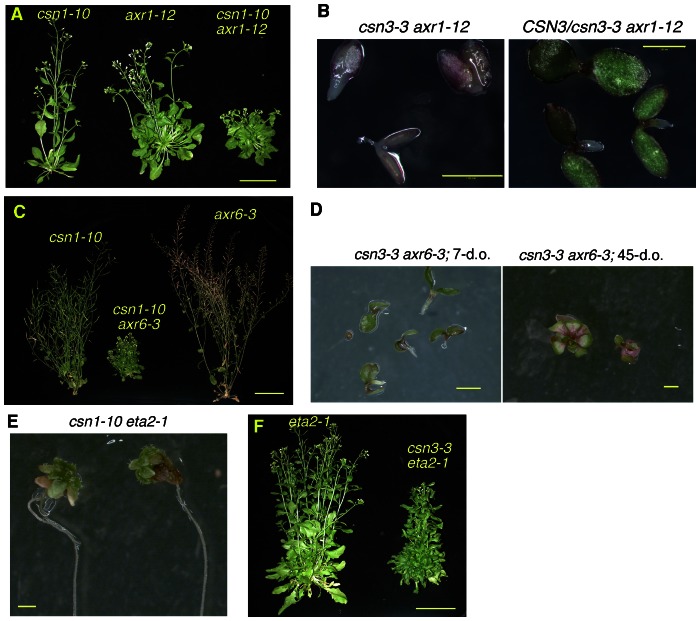
*csn3-3* and *csn1-10* exhibit distinct double mutant interactions. (**A**) Adult *csn1-10 axr1-12* double mutant exhibit a slightly more severe dwarf phenotype than either of the single mutants. Size bar = 4 cm. (**B**) *csn3-3* interacts with *axr1-12* synergistically, resulting in the seedling lethality. Right panel shows the seedling-lethal phenotype of heterozygous *csn3-3/CSN3* in the *axr1-12* background. Size bar = 1 mm. (**C–D**) While *csn1-10 axr6-3* plants are viable and complete the life cycle, *csn3-3* interacts with *axr6-3* synergistically, with double mutants exhibiting developmental arrest at the seedling stage. Size bars = 4 cm (**C**) and 1 mm (**D**), respectively. (**E–F**) *csn1-10* exhibits a stronger interaction with *eta2-1* than does *csn3-3*. As previously reported [43], *csn1-10 eta2-1* seedlings fail to develop past the early seedling stage. Size bars = 1 mm (**E**) and 4 cm (**F**), respectively.

We therefore crossed *axr1-12* plants with *csn1-10* and *csn3-3* to generate double mutants. The *axr1-12* mutation affects a subunit of the NEDD8 activating enzyme. This mutation confers a reduction in CUL1 neddylation and strong auxin response defects [16], [17], [61]. The interaction between *axr1-12* and *csn1-10* appeared additive, as double mutants exhibited a moderately more severe dwarf phenotype than the single mutant parents (**Figure 5A**). In sharp contrast, *csn3-3 axr1-12* doubles displayed a seedling-lethal phenotype (**Figure 5B**), indicating that *axr1-12* and *csn3-3* interact synergistically. Double mutant seedlings exhibited cotyledon morphogenic defects and lacked a basal pole similar to loss-of-function mutants of the *monopteros* (*mp*) auxin response factor and the dominant negative *axr6-1* and *axr6-2* alleles of *CUL1* [14], [62]. Furthermore, a slightly less severe phenotype was observed in *CSN3/csn3-3 axr1-12/axr1-12* seedlings (**Figure 5B**). A similar set of genetic interactions was observed when *csn1-10* and *csn3-3* were combined with *axr6-3*. *axr6-3* is a temperature-sensitive allele of *CUL1* that exhibits reduced CUL1 neddylation and severe auxin response defects [15]. While *csn1-10 axr6-3* plants were viable, flowered, and produced a few seeds, *csn3-3 axr6-3* plants exhibited arrested development, with virtually no root growth occurring even after 45 days of growth (**Figure 5C, D**).

In contrast to the double mutants with *axr1-12* and *axr6-3*, the phenotypic severity was reversed when *csn1-10* and *csn3-3* were combined with the *eta2-1* mutation. *eta2-1* is a missense mutation in CAND1, a cullin binding protein that mediates cycles of SCF complex assembly and disassembly [43], [52]. While the *eta2-1* mutation abolishes CUL1 binding activity and thus disrupts SCF cycling, it has no detectable effect on CUL1 neddylation. Whereas *csn1-10 eta2-1* double mutants failed to progress past the seedling stage [43], *csn3-3* only slightly enhanced the developmental defects of *eta2-1* (**Figure 5F**). Together, the highly differential genetic interactions conferred by the *csn1-10* and *csn3-3* mutations in combination with *axr1-12*, *axr6-3*, and *eta2-1* strongly suggest that these two mutations in CSN subunits affect distinct aspects of auxin signaling. This notion is consistent with our finding that only *csn1-10* exhibits defects in cullin deneddylation and SCF^TIR1/AFB^-mediated regulation of Aux/IAA protein stability.

### A novel CSN3 complex, but not the CSN holocomplex, is altered in *csn3-3* plants

The CSN deneddylase activity is catalyzed by the CSN5 subunit, in which the JAMM motif is the catalytic center required for the isopeptidase activity to remove NEDD8 from the cullin proteins [33], [44]. However, the proper assembly of the CSN holocomplex is required for deneddylase activity. Consequently, null mutations in any of the eight CSN subunits abolish both CSN assembly and deneddylase activity, and confer similar seedling-lethal phenotypes [23], [48]–[50], [55], [59].

**Figure 6:**
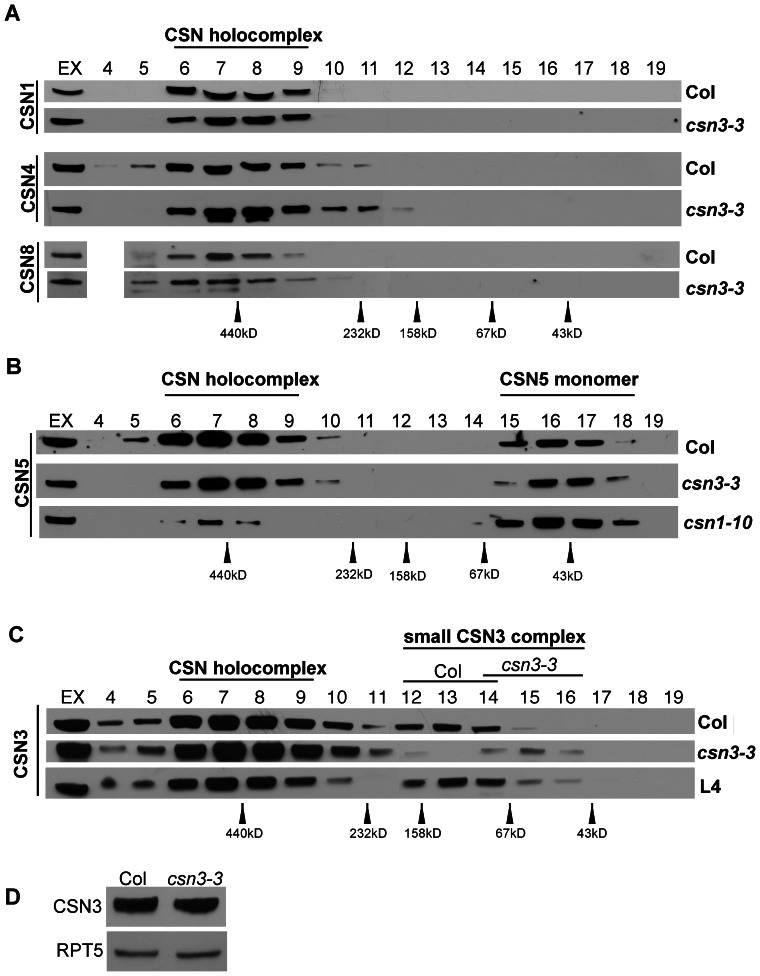
A small CSN3-containing complex, but not the CSN, is affected by the *csn3-3* mutation. Protein extracts from 7-d.o. Col, *csn1-10* and *csn3-3* seedlings were fractionated on a Superdex-200 gel filtration column and fractions (4 to 19) were collected and blotted with α-CSN1, α-CSN4, and α-CSN8 (**A**), α-CSN5 (**B**) and α-CSN3 (**C**). Fraction #4 was lost in the experiment examining CSN8. In (**C**), the assembly of a small CSN3-containing protein complex was observed in fractions 12-14. This complex was absent in *csn3-3* extracts, but was restored by introduction of a genomic *CSN3* transgene (L4 complementation line). Molecular mass standards are shown at the bottom of each panel. EX indicates the protein crude extract before gel filtration. (**D**) CSN3 protein level is unaffected by the *csn3-3* mutation. RPT5 is shown as a loading control.

Since *csn3-3* confers reduced auxin signaling but does not affect the deneddylase activity of the CSN, we examined whether the *csn3-3* mutation had any effect on CSN complex assembly. Protein extracts from Col and *csn3-3* seedlings were fractionated by gel filtration chromatography as previously described [43], and analyzed by immunoblotting with antibodies against several CSN subunits. With wild-type extracts, the CSN1, CSN4, and CSN8 subunits were detected primarily in high molecular mass fractions (#6-9) corresponding to 450-700 kD (**Figure 6A**), consistent with the previously observed molecular mass of the CSN holocomplex [23], [58]. No obvious differences from this pattern were detected with *csn3-3* extracts. Unlike other CSN subunits, prior studies in both plants and animals have demonstrated that the CSN5 subunit is present in both the CSN and as a monomer [34], [59], [63]. Consistent with these findings, we detected CSN5 in both high and low molecular mass fractions corresponding the CSN holocomplex and CSN5 monomer, respectively (**Figure 6B**). Again however, no differences between Col and *csn3-3* were observed. In contrast, the *csn1-10* mutation resulted in a clear shift in CSN5 fractionation, with the majority of CSN5 fractionating in the monomeric form (**Figure 6B**). This finding suggests that the *csn1-10* mutation impairs incorporation of CSN5 into the CSN holocomplex and is consistent with the reduction in cullin deneddylation we observe in this mutant.

Together with our finding that CSN deneddylase activity is unaltered in *csn3-3* mutants, the above gel filtration findings with CSN1, CSN4, CSN5, and CSN8 indicate that CSN holocomplex assembly is unaffected by *csn3-3*. Consistent with this conclusion, we found that the abundance of the *csn3-3* mutant protein is similar to wild-type, and the mutant protein also incorporates into the CSN holocomplex (**Figures 6C, D**). However, during our gel filtration studies with CSN3, we noticed an additional CNS3 elution peak centered around 130 kD in wild-type extracts (**Figure 6C** **, fractions 12-14**). Interestingly, this small CSN3-containing complex (sCSN3c) was absent in *csn3-3* seedlings, indicating that the *csn3-3* missense mutation specifically disrupts the formation of this complex. Furthermore, the sCSN3c was restored by expression of a *P_CSN3_:CSN3* transgene in *csn3-3* mutant plants (**L4 in** **Figure 6C**).

Our results suggest that sCSN3c represents a novel CSN3-containing complex and not an intermediate in CSN holocomplex assembly. First, a prior CSN subunit interaction mapping study concluded that the CSN3 subunit primarily contacts CSN1, CSN8, and probably CSN4 [64]. However, none of these subunits co-eluted with sCNS3c in our gel filtration experiments (**Figure 6A**). Likewise, no overlap in the elution profile of CSN5 with sCSN3c was observed (**Figure 6A, B**). Secondly, we tested the possibility that sCSN3c may be due to partial disassembly of the CSN that occurred during our *in vitro* fractionations. Fractions containing the CSN holocomplex were isolated and subjected to a second round of gel filtration and subsequently immunoblotted with α-CSN3 antibody (**Figure S3**). However, no CSN3 protein was detected in the low molecular mass fractions corresponding to sCSN3c, demonstrating that the CSN is stable under our conditions. Together, these findings suggest that CSN3 is the only canonical CSN subunit present in sCSN3c, although the possibility that subunits for which we do not have antibodies (CSN2/6/7) are sCSN3c components cannot be eliminated.

## Discussion

In plants, animals, and fungi, the CSN has a well-established role as a cullin deneddylase that regulates CRL ubiquitin-ligase activity [44]. Prior reports in Arabidopsis have demonstrated that reduced deneddylase activity in various *csn* mutants affects SCF^TIR1/AFB^ ubiquitin-ligase activity and consequently results in impaired auxin signaling [28], [35], [40]. In this study, we identified the *csn3-3* missense mutation as an enhancer of the auxin resistant root growth phenotype conferred by *tir1-1*. Consistent with diminished auxin signaling, *csn3-3* also enhanced the *tir1-1* lateral root development defect and conferred diminished auxin mediated expression of the *DR5:* and *BA3:GUS* reporters. While these findings were hardly surprising given the aforementioned studies of Arabidopsis CSN subunit mutants, we quite unexpectedly found that neither the deneddylation activity of the CSN nor the SCF^TIR1/AFB^-mediated regulation of Aux/IAA protein stability was affected by the *csn3-3* mutation. These findings clearly distinguish *csn3-3* from all previously characterized *csn* mutants.

**Figure 7:**
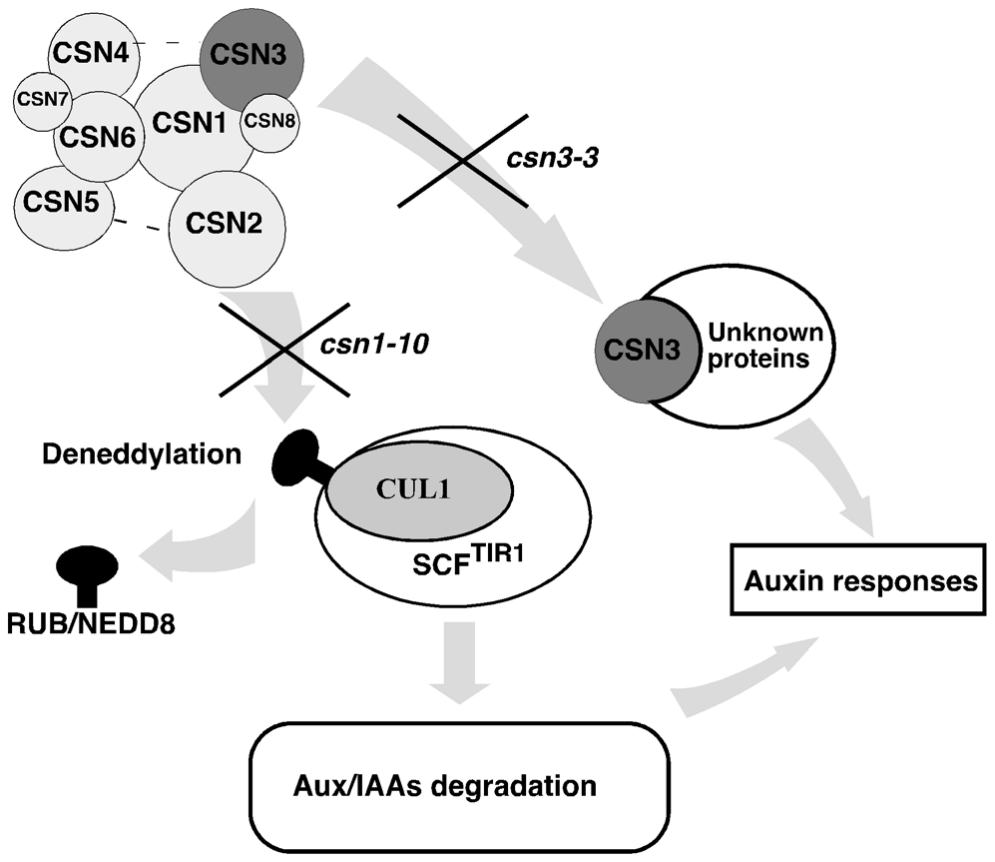
The CSN3 subunit plays multiple roles in auxin signaling. We hypothesize that in addition to its role in the CSN as a cullin deneddylase, the CSN3 subunit also regulates auxin signaling independently of the SCF^TIR1/AFB^ ubiquitin-ligase. This second regulatory mechanism may involve the small CSN3-containing complex whose assembly is disrupted by the *csn3-3* point mutation.

It could be argued that *csn3-3* is simply a weak mutation that does not confer sufficient defects to be detected in assays examining neddylation status of cullins or the SCF^TIR1/AFB^ activity. However, our comparison of *csn3-3* phenotypes to those of a second weak CSN subunit mutant, *csn1-10*, strongly argues against this possibility. *csn3-3* and *csn1-10* confer virtually identical dose-response curves in assays examining auxin-mediated inhibition of root growth, with both mutants exhibiting 50% growth inhibition at ∼50 nM 2,4-D. Likewise, both mutants enhance the *tir1-1* auxin resistant root growth and reduced lateral root development phenotypes to a similar extent. Furthermore, both *csn3-3* and *csn1-10* confer comparable reductions in auxin mediated expression of the *BA3*: and *DR5:GUS* reporters. Together, these findings indicate that *csn3-3* and *csn1-10* impair auxin signaling to a similar extent. However, whereas cullin deneddylation and Aux/IAA degradation were unaffected in *csn3-3* seedlings, both of these molecular defects were clearly apparent in *csn1-10* mutants. Prior studies have clearly demonstrated that the CSN3 subunit is required for CSN deneddylase activity [35], which we have confirmed with the *csn3-2* null allele. The *csn3-3* missense mutation however, confers a reduction in auxin response without affecting cullin deneddylation, suggesting that CSN3 plays a second role in auxin signaling in addition to its role in regulating the SCF^TIR1/AFB^ ubiquitin ligase (**Figure 7**).

The dramatically different double mutant phenotypes exhibited when the *csn1-10* and *csn3-3* mutations were combined with *axr6-3*, *axr1-12*, or *eta2-1* also indicate that *csn1-10* and *csn3-3* affect distinct aspects of auxin signaling. While *csn3-3* confers seedling lethality when combined with *axr6-3* or *axr1-12*, the *csn1-10* mutation does not. In contrast, *csn1-10*, but not *csn3-3*, confers a seedling arrest phenotype in the *eta2-1* background. Given that SCF^TIR1/AFB^ activity appears unaffected in *csn3-3* plants, it seems unlikely that the lethality of *csn3-3 axr1-12* and *csn3-3 axr6-3* seedlings is due to a further reduction in SCF^TIR1/AFB^ activity. Instead, we speculate that *csn3-3* specifically affects auxin signaling downstream or perhaps independently of SCF^TIR1/AFB^ (**Figure 7**), such that the combination of *csn3-3* with *axr6-3* or *axr1-12* causes auxin sensitivity to fall below the threshold required for early seedling development. Although it is unclear what these differential genetic interactions might mean mechanistically, it is interesting to note that both *axr6-3* and *axr1-12* result in a reduction in neddylated CUL1 [15], [16], [19], [37]. On the other hand, the *eta2-1* mutation has no effect on CUL1 neddylation status [52]. Rather, the *eta2-1* mutation abolishes the CUL1 binding activity of CAND1, resulting in the disruption of CAND1-mediated cycling of SCF complexes [43].

Consistent with the hypothesis that an SCF^TIR1/AFB^-independent pathway may regulate auxin signaling to control gene expression, like *csn3-3*, the previously described *ibr5* mutants of Arabidopsis also exhibit diminished auxin response without inhibiting SCF^TIR1/AFB^-mediated degradation of Aux/IAA proteins [60]. *IBR5* encodes a putative dual-specificity protein phosphatase. However, although Aux/IAA proteins are highly unstable in both *csn3-3* and *ibr5* mutants, *ibr5* seedlings exhibited reduced steady-state levels of the AXR3NT-GUS and IAA28-myc reporter proteins. In our analysis of *csn3-3* mutants, however, both of these reporter proteins were present at levels comparable to wild-type controls. Furthermore, unlike *csn3-3*, *ibr5* does not interact with *axr1* in a synergistic manner [60]. Together, these findings suggest that it is unlikely that *csn3-3* and *ibr5* share a common auxin signaling defect.

The fact that the *csn3-3* mutation did not diminish CSN deneddylase activity, yet conferred reduced auxin response phenotypes, suggests that this mutation defines a novel function for CSN3. Therefore, a major question is whether *csn3-3* defines a new role in auxin signaling for the CSN holocomplex or a distinct CSN3-containing complex. While deneddylation is the only known biochemical activity of the CSN itself, additional activities including deubiquitinylating and protein kinase activities have been reported to be associated with the CSN [44], [65], [66]. Furthermore, in animal systems some CSN subunits have been found to be DNA associated and suggested to regulate transcription [67], [68]. Also, whether or not all CSN subunits function solely within the CSN holocomplex is unclear. On one hand, null mutations in any of the eight Arabidopsis subunits confer identical seedling-lethal phenotypes [23] and transcription profiles [35], suggesting that each subunit only functions within the CSN. However, it is possible that CSN subcomplexes or individual subunits have additional functions that are masked by the early seedling lethality of these null mutants. Consistent with this possibility, fission yeast *csn1* and *csn2* mutations confer DNA replication defects whereas other subunit mutations do not [25]. Similarly, while both Drosophila *csn4^null^* and *csn5^null^* mutants are embryo-lethal, these two mutants exhibit distinct developmental arrest phenotypes [63], [69] and differentially affect gene expression [70].

Given that *csn3-3* confers no apparent defects in cullin deneddylation, SCF^TIR1/AFB^ activity, or CSN holocomplex assembly, but does specifically abolish the ∼130 kD sCSN3c complex, we hypothesize that a defective sCSN3c may be the basis of the auxin signaling defects displayed by *csn3-3* mutant plants (**Figure 7**). Consistent with this possibility, expression of a *P_CSN3_:CSN3* transgene in *csn3-3* mutant plants restored both the auxin response defects (**Figures 1C** **,** **2E**) and the sCSN3c complex (**Figures 6C**). Prior studies have reported CSN subunits in complexes smaller than the CSN holocomplex. While some support has been presented for CSN5 functioning autonomously of other CSN subunits [71], [72], these smaller CSN complexes have generally been proposed to be mini-CSN complexes containing several, but not all subunits [64]. Whether these represent intermediates in CSN holocomplex assembly or functionally distinct complexes is uncertain. Interestingly, one study examining these mini-CSN complexes from animal cells by non-denaturing polyacrylamide electrophosesis detected CSN3-containing complexes that appeared to lack other CSN subunits [71]. In Arabidopsis, Rubio et al. [73] also detected, but did not discuss or characterize, small CSN3-containing complexes.

Recent analyses of CSN subunit interactions using *in vitro* reconstituted human CSN subunits suggest that the CSN consists of two symmetrical modules; CSN1/2/3/8 and CSN4/5/6/7 [64]. With this subunit topology, CSN3 would directly interact with CSN1/8 and possibly CSN4, which is consistent with prior two-hybrid studies [74]. However, our gel filtration analysis indicates that neither CSN1/4/8 nor CSN5 are components of the sCSN3c complex. While we cannot eliminate the possibility that CSN2/6/7 are sCSN3c components, this seems unlikely given these prior findings. Thus, together with our finding that CSN holocomplex assembly is unaffected by *csn3-3*, we hypothesize that sCSN3c represents a novel complex rather than a mini-CSN complex. Identification of the other components within sCSN3c may provide crucial information into what role this complex might play in auxin signaling. Furthermore, since the residue affected by the *csn3-3* missense mutation is extremely highly conserved across eukaryotes, it seems likely that sCSN3c function may be similarly conserved.

## Materials and Methods

### Plant Materials and Growth Conditions

All Arabidopsis lines used in this study are in the Col-0 ecotype. Seeds were sterilized by 30% bleach + 0.1% Triton-X100 for 10 min and were stratified at 4°C for 1-4 days. Seedlings were grown under sterile conditions on vertically oriented ATS nutrient medium [61] under long-day conditions. Adult plants were grown in soil under long-day conditions at 20°C. The *tir1-1, csn1-10*, *csn3-2* (SALK_106465), *eta2-1*, *axr6-3*, and *axr1-12* mutants have been described previously [35], [43], [75]. The *BA3:GUS* [57], *DR5:GUS* [3], *HS:AXR3NT-GUS* [4], and *P_IAA28_:IAA28-myc* [60] transgenes were introduced into the *csn3-3* and *csn1-10* backgrounds by crossing. For construction of double mutant and reporter lines, the *csn3-3* mutation was genotyped using a CAPS marker for PCR products generated with primers Ex7F (5′-CAACGACGGGAAGATTGGTG-3′) and Ex8R (5′- GCCTCCTTAGCATTACCAAG-3′). When digested with *Sty* I, the 289 bp *CSN3* PCR product is cleaved into 163 and 126 bp fragments, whereas the *csn3-3* mutation abolishes the *Sty* I recognition site. The *eta2-1*, *axr1-12*, and *axr6-3* mutations were confirmed by sequencing PCR products spanning the mutation sites.

For root growth assays, 5-d.o. seedlings were transferred to ATS medium supplemented with various concentrations of 2,4-D, and root growth was measured after an additional 4 days. Percent inhibition was calculated by dividing the average growth on auxin media by the average growth on ATS control media and subtracting this ratio from 100%. For measuring IAA-induced root growth inhibition, 6-d.o. seedlings were transferred to freshly made IAA plates and were grown under long-day illumination through yellow long-pass filters to slow indolic compound breakdown. Protein extractions for gel filtration and western experiments were made from 7 - 10-d.o. seedlings grown in liquid ATS medium on a shaker at 20°C.

### Complementation

The *CSN3* genomic DNA construct was composed of the *CSN3* coding region together with a 1.3 kb fragment upstream of the start codon plus a 600bp fragment downstream of the stop codon. The whole sequence was amplified from BAC clone F18022 (5′-GGGGACAAGTTTGTACAAAAAAGCAGGCTCCTTTGATGGCGCCATGGTGG-3′ and 5′-GGGGACCACTTTGTACAAGAAAGCTGGGTCGTATGGAAACATGTGATAACC) and Gateway cloned into the vector pEarleyGate301 [76]. The construct was then transformed into *csn3-3[BA3:GUS]* plants using *Agrobacterium tumefaciens* strain GV3101 according to standard procedures [77]. Two independent T3 homozygous lines (L2 and L4) were used for the root growth and GUS assays to assess complementation.

### GUS Histochemical Staining

*BA3:GUS* and *DR5:GUS* assays were conducted as described previously with slight modifications [52]. 6-d.o. seedlings grown vertically on ATS plates were transferred into liquid ATS medium supplemented with 0.5 µM 2,4-D for 12 h (*BA3:GUS*) and 4 h (*DR5:GUS*) before GUS staining. For *HS:AXR3NT-GUS* assays, 6-d.o. Col, *csn1-10* and *csn3-3* seedlings homozygous for the reporter construct were heat-shocked for 2 h at 37°C to induce expression of the transgene. Seedlings then were transferred to 20°C medium with 1 µM 2,4-D for 20 min before GUS detection. Quantitative measurements of β-glucuronidase activity were conducted as described previously [4] with modifications: after heat shock for 2 h at 37°C, seedlings were transferred to 20°C liquid ATS medium for 15 min. Seedlings were then moved into medium supplemented with 1 µM 2,4-D, sampled at 0, 20, 40 and 60 min thereafter and stored in liquid nitrogen until protein extraction. Frozen seedlings were homogenized by a laboratory vibration mill (Mixer-Mill; Qiagen, cat. no. MM300) in extraction buffer (50 mM KPO_4_ pH 7, 0.1% triton X-100, 10 mM β-mercaptoethanol, 10 mM EDTA). Samples were then centrifuged to remove debris and the protein level of each sample was measured. Protein extracts were mixed with an equal volume of extraction buffer plus 4 mM MUG (4-methylumbelliferyl-B-D-glucoronide), incubated at 37°C overnight, and the fluorometric signal measured using a BioTek FL600 Fluorescence Microplate Reader (Winooski, VT) as per manufacturer's instructions (excitation at 360 nm, emission at 460 nm). Activity was calculated as fluorometric units per μg protein and were normalized as the percentage of the starting point (0 min).

### IAA28-myc degradation assay

Experiments were done as described previously [60] with modifications. 7-d.o. light-grown seedlings of Col, *csn1-10* and *csn3-3* carrying the IAA28-myc construct were removed from ATS plates and floated in liquid ATS supplemented with 0.5 µM IAA. At the indicated time points, roots were excised and homogenized for protein extraction.

### Antibodies and Immunoblot Analysis

The CUL1 antibody has been described previously [9] and the CUL4 antibody was obtained from Dr. Xing Wang Deng (Yale U.). The quantification of CUL1-NEDD8:CUL1 ratios was performed in Image J. Antibodies against the CSN1, CSN3, and CSN8 subunits were raised by immunizing New Zealand white rabbits (Cocalico Biological, Reamstown, PA) with recombinant 6xHis-CSNx protein purified from *E. coli* using standard protocols [9]. Crude sera were subsequently affinity purified against nitrocellulose-bound antigens [78]. The CSN4, CSN5 and Rpt5 antibodies were purchased from BIOMOL Int'l, L.P./Enzo Life Sciences. Monoclonal α-myc 9E10 antibody was purchased from Covance and used as recommended. For IAA28-myc, cullins and CSN subunit immunoblotting, protein extracts were prepared from 7- to 10-d.o. seedlings (or seedling roots for IAA28-myc assay) in protein extraction buffer (50 mM Tris-HCl pH 7.5, 150 mM NaCl, 0.5% NP40, 1 mM DTT, 1 mM phenylmethylsulfonyl fluoride (PMSF), and 1X Protease Inhibitor Cocktail Kit (Thermo)). 50 µg of protein were loaded in each lane and separated by 10% SDS-PAGE (cullins and CSN subunits) or 4∼12% NuPAGE® Bis-Tris Gel (Invitrogen). For gel filtration, proteins of each fraction were concentrated with StrataClean™ Resin (Stratagene) and separated on 4∼12% NuPAGE® Bis-Tris Gel (Invitrogen), blotted, and used for western detection.

### Gel Filtration Chromatography

Experiments were conducted as described previously [43]. In brief, 7-d.o. seedlings grown in liquid ATS medium were homogenized in extraction buffer (50 mM Tris-HCl, pH 7.5, 150 mM NaCl, 1 mM EDTA, 10% glycerol, 10 mM MgCl_2_, 0.5 mM NaN_3_, 1 mM DTT, 1 mM PMSF, and 1X Protease Inhibitor Cocktail Kit (Thermo)). Homogenates were centrifuged twice for 10 min at 4°C to remove debris. Supernatants were then filtered though a 0.45 µm HT Tuffryn® Membrane (Pall). 600 µg of total protein was fractionated through a Superdex 200 10/300 GL column (Amersham/GE Healthcare). After loading the sample, proteins were eluted in filtered and degassed extraction buffer at a flow rate of 0.2 mL/min. 0.5 mL fractions were collected after the 6 mL void volume was reached. All procedures were carried out at 4°C.

## Supporting Information

Figure S1 **IAA induced** ***BA3:GUS*** **expression is reduced by the** ***csn1-10*** **and** ***csn3-3*** **mutations to a similar extent.** 6-d.o. transgenic Col, *csn1-10* and *csn3-3* seedlings carrying the *BA3:GUS* reporter were treated with 1 µM IAA for 3 h before histochemical staining for β-glucuronidase activity.
(TIF)

Figure S2 **α-myc western detection of the IAA28-myc fusion protein.** The P_IAA28_:IAA28-myc reporter was introduced into the *csn1-10* and *csn3-3* backgrounds by crossing. Protein extracts were made from 7-d.o. seedling roots treated with IAA for the indicated time. RPT5 was used as a loading control.
(TIF)

Figure S3 **sCSN3c is not a breakdown product of the CSN holocomplex during the** ***in vitro*** **fractionation.** Fractions (#5∼9) of the first gel filtration run using Col seedling protein extracts were isolated and injected into the column for a second round of gel filtration. CSN3 western detection was conducted using fractions from the 2nd gel filtration. No CSN3 was detected in the sCSN3c fractions.
(TIF)
