# Supplementary figures and images for: The eta7/csn3-3 Auxin Response Mutant of Arabidopsis Defines a Novel Function for the CSN3 Subunit of the COP9 Signalosome
Source: PLoS One. 2013 Jun 7;8(6):e66578. doi: 10.1371/journal.pone.0066578 (PMC3676356; doi:10.1371/journal.pone.0066578)

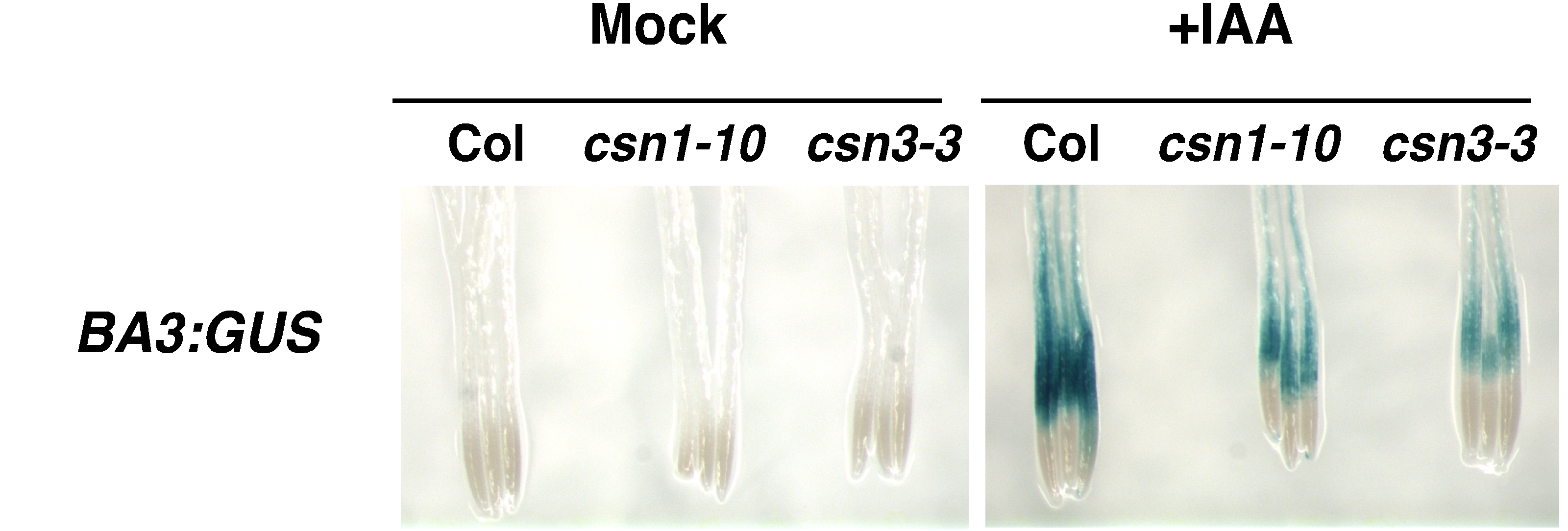

Supplement: Figure S1 — IAA induced BA3:GUS expression is reduced by the csn1-10 and csn3-3 mutations to a similar extent. 6-d.o. transgenic Col, csn1-10 and csn3-3 seedlings carrying the BA3:GUS reporter were treated with 1 µM IAA for 3 h before histochemical staining for β-glucuronidase activity. (TIF) [file pone.0066578.s001.tif]

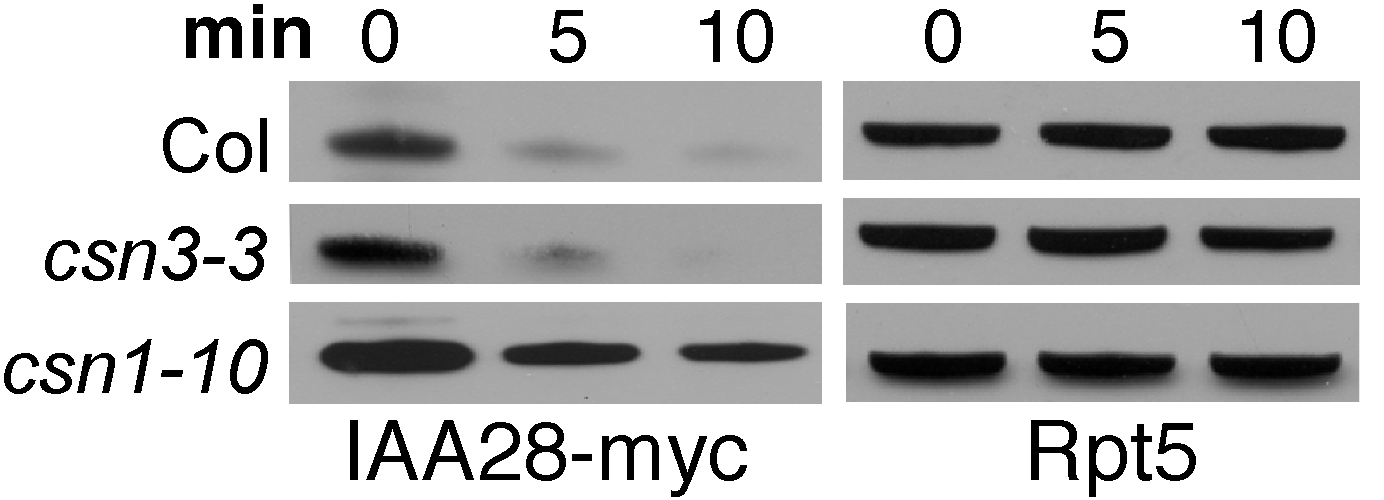

Supplement: Figure S2 — α-myc western detection of the IAA28-myc fusion protein. The PIAA28:IAA28-myc reporter was introduced into the csn1-10 and csn3-3 backgrounds by crossing. Protein extracts were made from 7-d.o. seedling roots treated with IAA for the indicated time. RPT5 was used as a loading control. (TIF) [file pone.0066578.s002.tif]

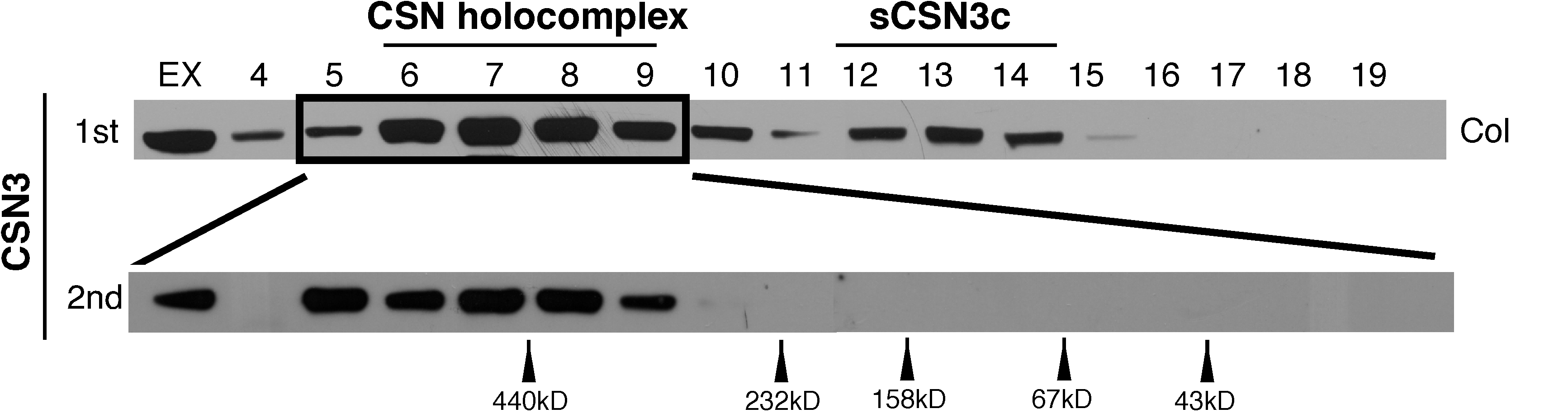

Supplement: Figure S3 — sCSN3c is not a breakdown product of the CSN holocomplex during the in vitro fractionation. Fractions (#5∼9) of the first gel filtration run using Col seedling protein extracts were isolated and injected into the column for a second round of gel filtration. CSN3 western detection was conducted using fractions from the 2nd gel filtration. No CSN3 was detected in the sCSN3c fractions. (TIF) [file pone.0066578.s003.tif]
